# Damage Mechanism of GaN HEMT and Failure Analysis of Power Amplifier Under High-Altitude Electromagnetic Pulse

**DOI:** 10.3390/mi17091085

**Published:** 2026-09-16

**Authors:** Lu Sun, Haolin Wu, Jin Tian, Keke Bai

**Affiliations:** State Key Laboratory of Electromechanical Integrated Manufacturing of High-Performance Electronic Equipment, School of Electro-Mechanical Engineering, Xidian University, Xi’an 710071, China; sunl@mail.xidian.edu.cn (L.S.); 25041212726@stu.xidian.edu.cn (H.W.); 18864697856@163.com (K.B.)

**Keywords:** GaN HEMT, power amplifier, High-altitude Electromagnetic Pulse, damage effect

## Abstract

With the growing complexity of electromagnetic environments, electronic systems suffer from prominent strong electromagnetic interference in practical service. As a key component implementing power amplification and transmission in communication systems, interference and damage effects of a GaN HEMT power amplifier under High-altitude Electromagnetic Pulse (HEMP) directly affect the regular operation of systems. In this paper, a physical device model and an injection source model are built first; injection simulations of different HEMP pulses are adopted to analyze internal temperature and current density distributions, predicting vulnerable positions of the device under gate injection. A GaN HEMT power amplifier based on CGH40010F is then established to investigate the failure mechanism under HEMP injection and the damage effect of different pulse parameters. An injection experiment system is conducted according to HEMP pulse standard; results indicate that power amplifier failure stems from GaN HEMT device destruction. The damage evolution is tightly associated with injected energy accumulation, and the gate–source channel is the susceptible region for GaN HEMT under gate injection. These conclusions can provide important references for the protective design of GaN HEMT power amplifiers.

## 1. Introduction

**Figure 1:**
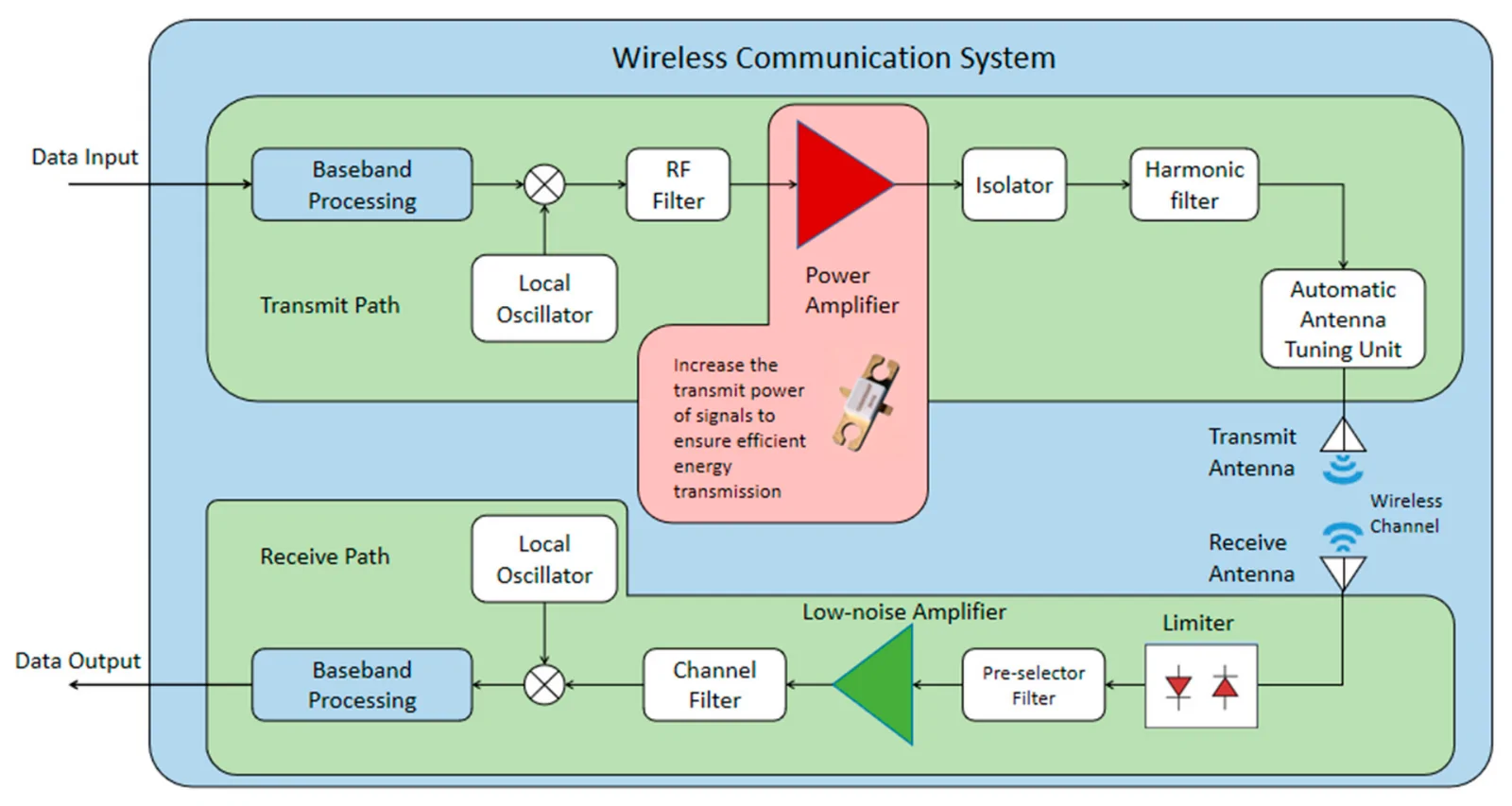
Block diagram of the wireless communication system.

HEMP is characterized by high peak amplitude, steep rise time, wide spectrum, and strong destructive capability [1], which can couple into Radio Frequency (RF) systems through multiple paths and bring great threats to circuits and devices. Figure 1 shows the architecture of a wireless communication system, which mainly comprises the transmit path, receive path and wireless channel. The power amplifier is located in the transmit path of the RF system and is used to amplify RF signals for long-distance transmission and high-quality radiation, which is the critical component of the RF front-end. HEMP can couple into RF systems via antennas, transmission lines, power supply networks and other paths, impacting transceivers and their critical circuits [2,3]. As the core component, the power amplifier is prone to performance degradation, functional failure and even breakdown damage under HEMP irradiation, which severely undermines stable system operation.

Power amplifiers can be categorized by their internal active devices into vacuum tube power amplifiers, bipolar transistor power amplifiers, silicon-based MOSFET power amplifiers, Gallium Arsenide (GaAs)-based power amplifiers, Gallium Nitride High Electron Mobility Transistor (GaN HEMT) power amplifiers and so on. Among them, GaN HEMT has become an important device foundation for the design of new-generation RF power amplifiers by virtue of its wide bandgap, high breakdown electric field, high electron saturation drift velocity and excellent high-temperature operation capability [4,5,6]. However, GaN HEMT exhibits obvious vulnerability under HEMP injection. Exposure to HEMP may trigger abnormal device operation and partial structural burnout, which further impairs the normal function of power amplifiers and even reduces the operating efficiency of RF communication systems. Therefore, exploring the damage effects of GaN HEMT and its power amplifiers under HEMP is of great significance for improving the reliability of RF systems.

In recent years, extensive studies have been conducted on the response characteristics, damage processes, and failure mechanisms of RF systems under high-power electromagnetic pulse exposure, as well as the damage mechanisms of semiconductor devices. In 2018, Yan et al. combined Technology Computer Aided Design (TCAD) simulation with injection experiments to investigate the microwave damage of HEMT low-noise amplifiers under different drain bias conditions. The results showed that the damage power threshold changed little between normal-bias and zero-bias conditions, whereas the bias condition significantly affected the output response characteristics [7]. In 2020, Sangwan et al. studied electromagnetic failures of GaN HEMT high-frequency power amplifiers. They found that intensified local electromagnetic fields under high power could trigger device failure at rated input, revealing the electromagnetic failure characteristics from device and layout field distribution [8]. In 2024, Wang et al. investigated the high-power electromagnetic pulse-induced failure of a GaN HEMT power amplifier module. Stepwise pulse injection experiments were performed to determine the performance degradation and failure thresholds. Numerical analysis further identified the GaN HEMT as the most vulnerable component in the module [9].

Overall, studies on HEMP-induced damage in GaN HEMT and its power amplifiers remain limited and lack systematic investigation. In this study, the damage effects of GaN HEMT and its power amplifiers under HEMP exposure are systematically investigated through combined simulation and experiments. The results provide a theoretical basis for optimizing device structures and protection circuits and ensuring the stable operation of radar, satellite, military communication and industrial radio frequency equipment under extreme electromagnetic environments.

## 2. Damage Analysis of GaN HEMT Under HEMP Injection

In this section, a GaN HEMT device model and a HEMP injection source model are constructed first, and HEMP gate injection simulations for the GaN HEMT are subsequently carried out. By tuning the parameters of the injection source, variations in the internal physical parameters of the GaN HEMT are analyzed, which reveals the device’s damage effects when subjected to HEMP injection.

The CGH40010F GaN HEMT manufactured by Wolfspeed (Durham, NC, USA) is selected as the research object, and this device is used in all subsequent simulations and experiments. For the subsequent power-amplifier design, the L-band is selected as the primary operating frequency band. It occupies the frequency range of 1–2 GHz in the radio spectrum, features strong penetration capability, and is widely used in satellite communication, radar and other applications. The CGH40010F operates over DC-6 GHz and covers the entire L-band. Its saturated output power, small-signal gain and other parameters satisfy the power amplifier design targets, which is the primary reason for choosing this device. Moreover, the manufacturer supplies the cross-sectional structural data of the chip and the ADS circuit model for subsequent device modeling and experimental validation.

### 2.1. Structural Model

**Figure 2:**
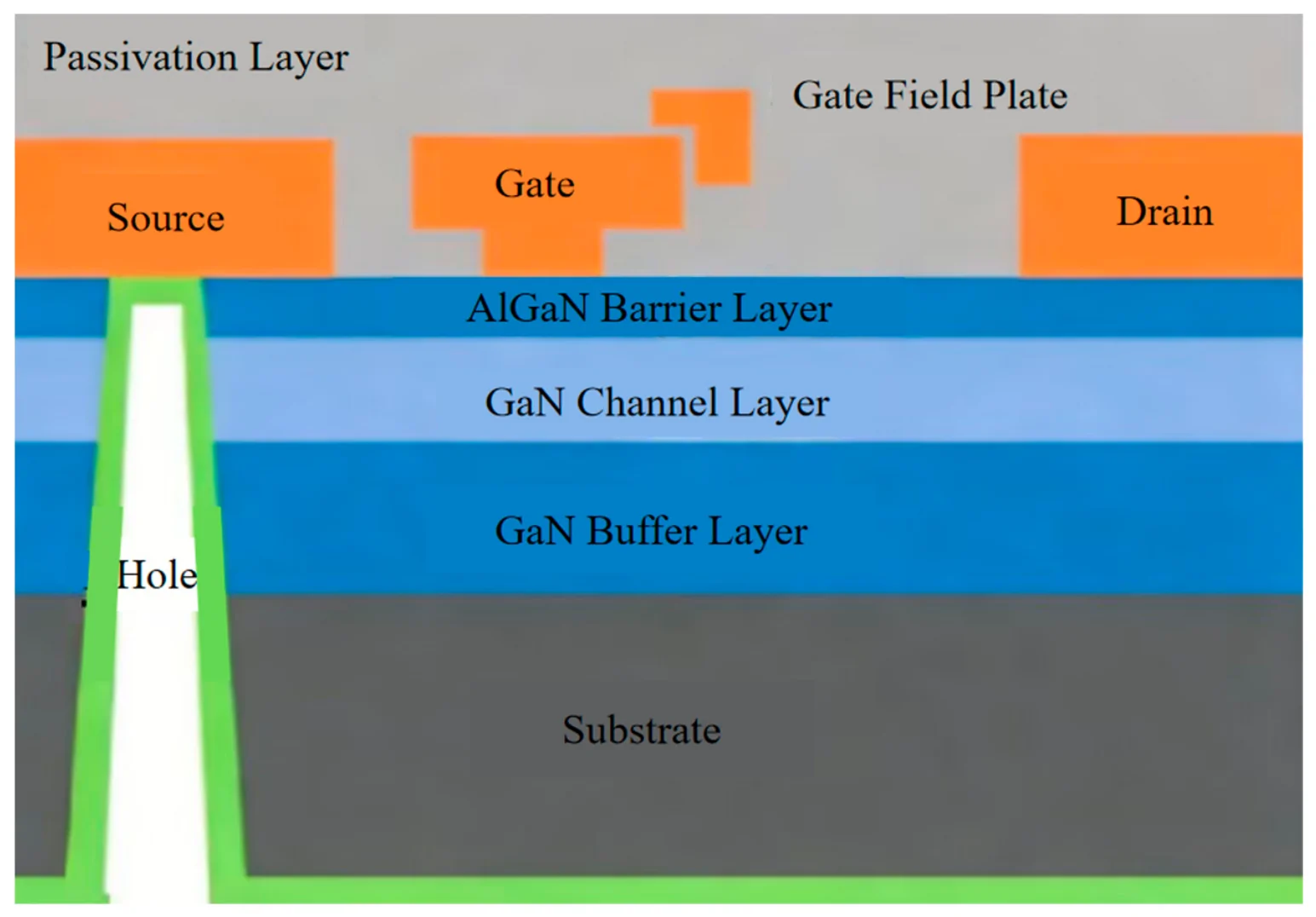
Cross-sectional view of the GaN HEMT.

Figure 2 shows the cross-sectional structure of the GaN HEMT established using Silvaco TCAD [10], and Table 1 summarizes its key parameters. As shown in Figure 2, the device consists of a substrate, multiple semiconductor epitaxial layers, metal electrodes, and a passivation layer. From bottom to top, the epitaxial structure comprises a GaN buffer layer, a GaN channel layer, and an AlGaN barrier layer. A via hole is formed in the source region to connect the source electrode to the underlying conductive layer. The source, drain, and field-plate gate are located beneath the passivation layer, while the two-dimensional electron gas formed at the AlGaN/GaN heterojunction serves as the conductive channel.

### 2.2. Numerical Model

**Figure 3:**
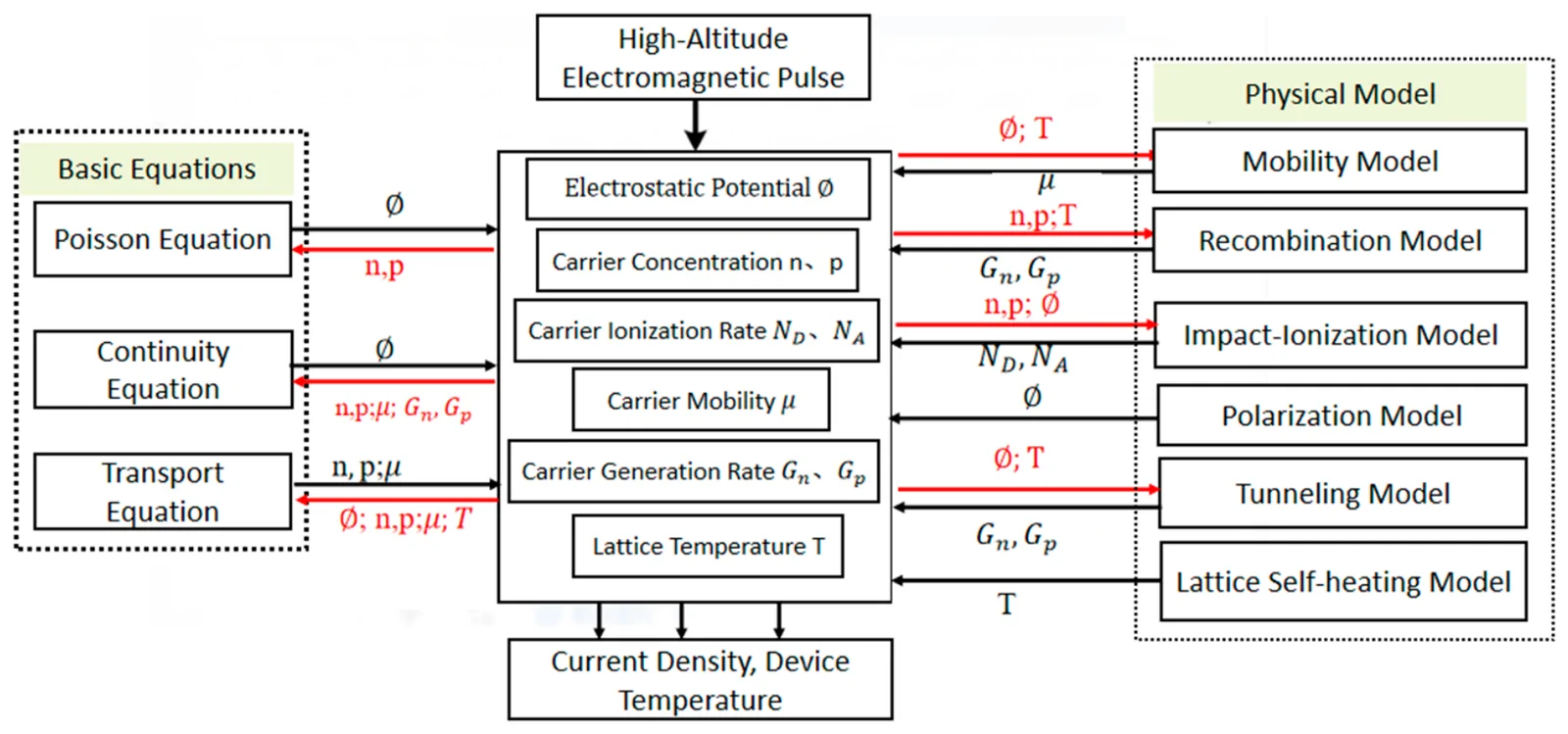
Coupling of the governing equations and physical models.

Figure 3 illustrates the coupling relationships among the governing equations and physical models used in the GaN HEMT simulation [11,12]. The Poisson equation describes the relationship between the electrostatic potential and charge distribution, the continuity equations ensure carrier conservation, and the transport equations describe carrier transport. The physical models account for practical device behaviors, including carrier mobility characteristics, carrier recombination and generation, impact ionization effect, and so on. By coupling these equations and models, the multiphysics behavior of GaN HEMT under HEMP exposure, including carrier dynamics, electrothermal effects, and breakdown mechanisms, can be accurately simulated. The distributions of the electric field, current density, and temperature are obtained to predict the transient response and failure behavior of the device.

### 2.3. Circuit Model

#### 2.3.1. Injection Source Model

Building an injection source is the prerequisite for simulation. This work constructs the injection source model complying with GJB 8848 [13], MIL-STD-188-125-2 [14] and existing references.

**Figure 4:**
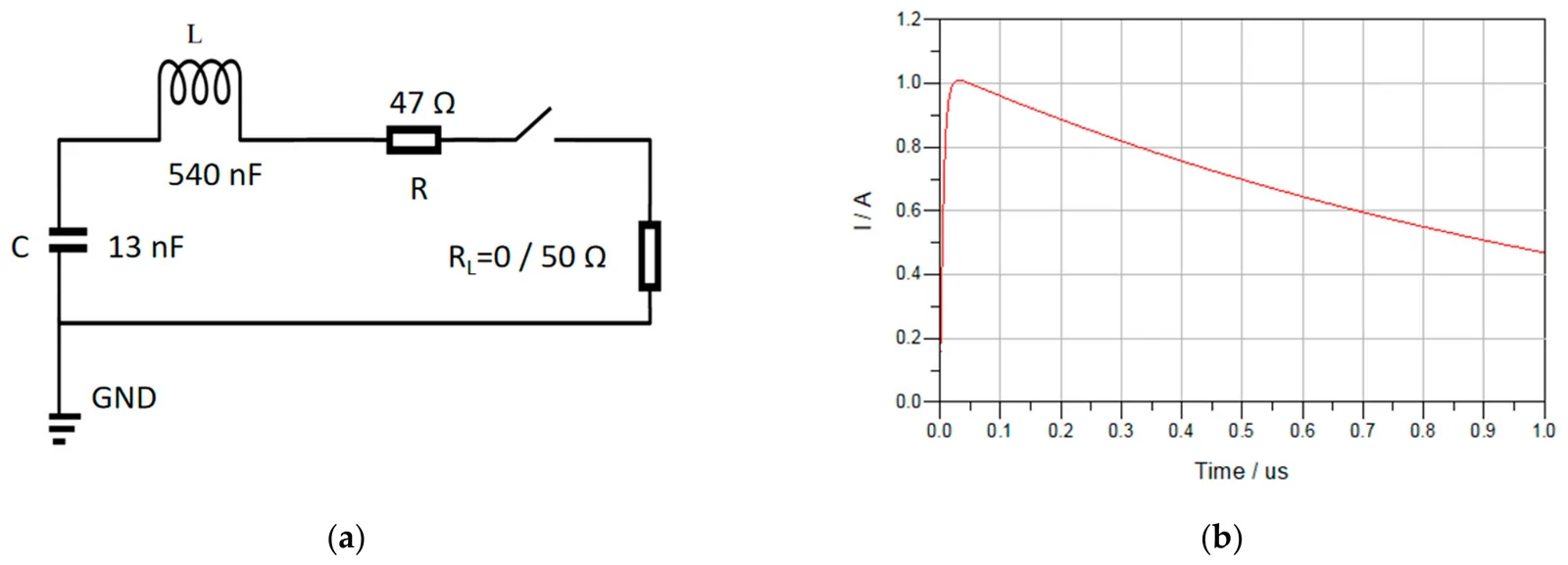
Equivalent circuit and coupled waveform. (**a**) 20/500 ns HEMP injection source equivalent circuit. (**b**) Coupled waveform of 20/500 ns HEMP.

The injection source model relies on the RLC equivalent circuit plotted in Figure 4a. The capacitor stores energy, with its initial voltage governing the pulse peak amplitude. The inductor regulates the pulse rise time. Resistor *R* eliminates circuit oscillation and modulates pulse width, while *R_L_* represents the equivalent matching impedance of coupling paths [15,16].

The modeling process adopts the 20/500 ns (the pulse rise time from 10% to 90% of the peak amplitude is 20 ns, and the time interval between the two half-peak crossing points is 500 ns) waveform as an example. The coupling impedance *R_L_* is set to 0 Ω. The mathematical expression for the HEMP waveform is defined as [17]

(1) $$U(t)=U_{P}(e^{-\beta t}-e^{-\alpha t})$$

At the peak instant, the time derivative of the function is zero, and the peak time can be solved as

(2) $$t_{p}=\frac{ln(\alpha /\beta )}{\alpha -\beta }$$

According to the definitions of the rise time and half-maximum width, two constraint equations can be obtained:

(3) $$F_{1}(\alpha ,\beta )=t_{90}-t_{10}-t_{r}=0$$

(4) $$F_{2}(\alpha ,\beta )=t_{w\_2}-t_{w\_1}-t_{w}=0$$

where *t_r_* is the pulse rise time, and *t_w_* is the pulse width. *t*_90_ is the time at which the pulse reaches 90% of its peak value, and *t*_10_ is the time at which the pulse reaches 10% of its peak value. *t_w__*_1_ denotes the time at which the waveform first reaches the half-peak value, and *t_w__*_2_ denotes the time of the second half-peak crossing. Among these parameters, *t*_90_, *t*_10_, *t_w__*_1_ and *t_w__*_2_ can all be expressed as implicit functions of α and β, and the analytical expressions of the four parameters are obtained by adopting the bisection method. The Newton–Raphson iterative method is employed to acquire the exact solutions: $\alpha =8.702\times 10^{7}\;s^{-1}$, $\beta =1.670\times 10^{6}\;s^{-1}$. The characteristic equation of the circuit is constructed as

(5) $$s^{2}+\frac{R}{L}s+\frac{1}{LC}=0$$

The characteristic roots are set as −α and −β, hence

(6) $$\left\{\begin{array}{c} \alpha +\beta =\frac{R}{L}\\ \alpha \beta =\frac{1}{LC} \end{array}\right.$$

*R* is treated as a free design variable adjustable based on simulation and experimental outcomes. With *R* set to 47 Ω, *C* = 13 nF and *L* = 540 nH are obtained.

In practical environments, *R_L_* (the equivalent matching impedance of coupling paths in Figure 4a) changes the total circuit damping and distorts the coupled HEMP waveform relative to the original radiating source. Figure 4b illustrates the coupled waveform of the 100 V peak, 20/500 ns HEMP under a 50 Ω source load. A 50 Ω equivalent matching impedance is adopted throughout all follow-up simulations and experiments in this study.

This identical method adjusts pulse peaks by changing the capacitor’s initial voltage and modulates rise time and pulse width via tuning RLC component values. Table 2 summarizes the required RLC parameters for 10/100 ns, 20/500 ns and 50/1000 ns HEMP waveforms.

#### 2.3.2. Gate-Injected HEMP Excitation Circuit for GaN HEMT

**Figure 5:**
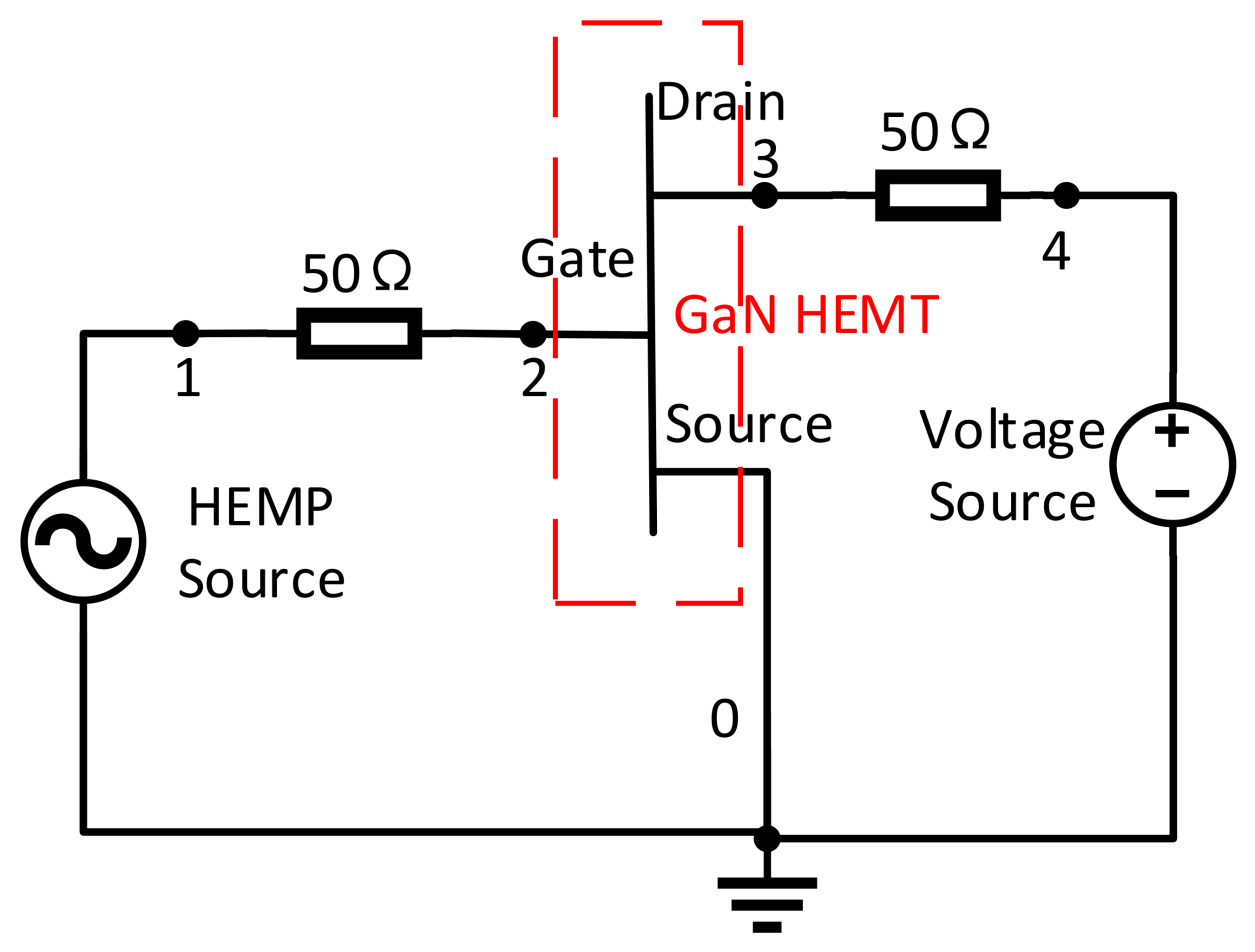
Simulation of gate HEMP injection into the GaN HEMT.

Figure 5 shows the HEMP injection simulation circuit for the GaN HEMT [18]. As indicated in the component datasheet, the CGH40010F is a depletion-mode field-effect transistor operating on a 28 V DC supply. A 50 Ω bias resistor is connected to the drain with the source grounded, while the series 50 Ω resistor at the gate input offers isolation and impedance matching, and the device stays conductive prior to pulse injection. HEMP propagates through the 50 Ω resistor to the gate and charges the gate-source capacitance, creating high-voltage transient overshoots superimposed on the DC bias. The waveform of the HEMP source is the coupled waveform obtained in Section 2.3.1.

Prior to simulation execution, the key parameters are configured in the TCAD. The mesh is refined to a minimum spacing of 0.001 μm at the AlGaN/GaN heterojunction interface, 0.01 μm at the gate edge region, and 0.2 μm in the substrate region. The transient simulation is performed with a total duration of 1000 ns and a time step of 0.01 ns. The HEMP pulse is injected at the gate, while the drain voltage steps from 0 V to 28 V within 2 ps. The thermal boundary condition is set to 300 K at the top of the device, and the peak temperature monitoring limit is set to the melting point of GaN, 1973 K. The device is defined to be burned out when its internal temperature reaches this temperature. All other parameters are set to their default values.

### 2.4. TCAD Simulation Results and Discussion

#### 2.4.1. Electrothermal Damage Analysis of GaN HEMT Under Gate Injection

**Figure 6:**
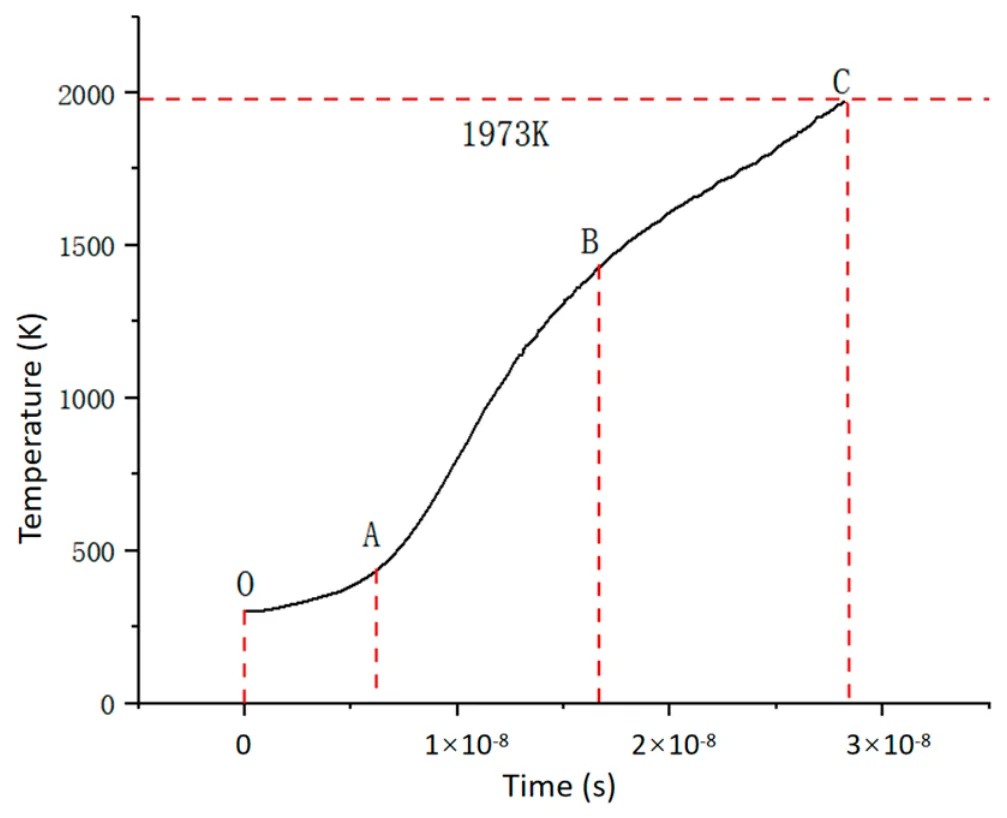
Time evolution of peak temperature in GaN HEMT under gate injection.

The circuit is built as illustrated in Figure 5, and the injection source is adjusted to output a HEMP pulse with a peak voltage of 300 V and 20/500 ns parameters for simulation, with the resulting device temperature–time curve presented in Figure 6.

As shown in Figure 6, the temperature rise process of the device can be divided into three stages, exhibiting a trend of “slow increase–rapid rise–gradual saturation” in heating rate. This electrothermal damage behavior of GaN HEMT can be explained by analyzing the evolution of multiple internal physical quantities during the heating process.

**Figure 7:**
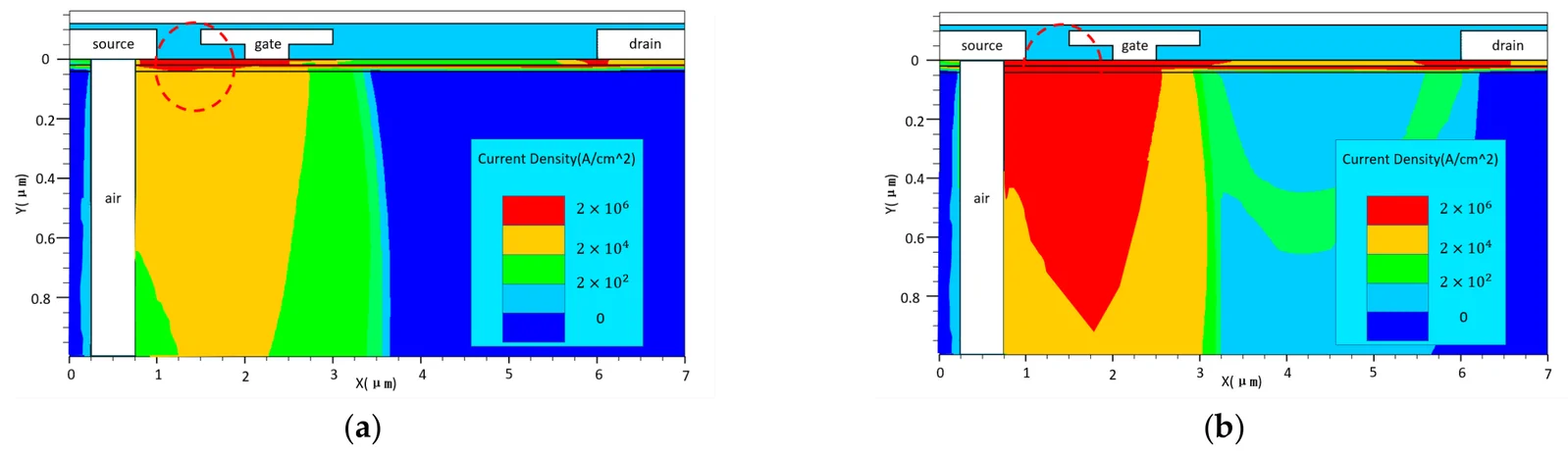
Current density in GaN HEMT under gate injection at different times. (**a**) 10 ns; (**b**) 20 ns.

**Figure 8:**
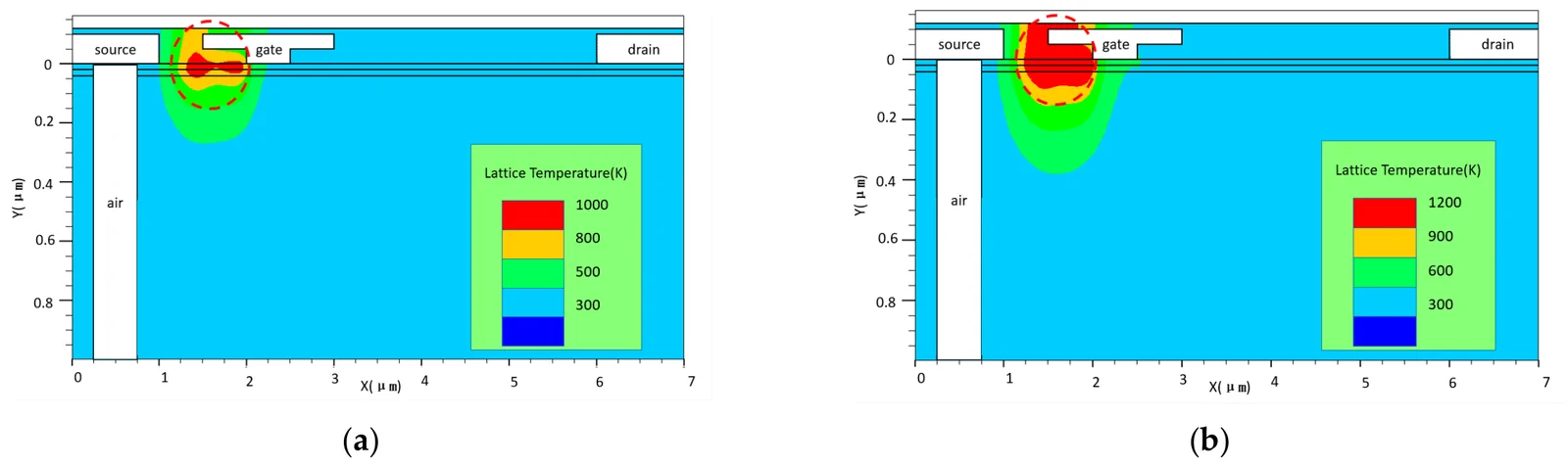
Temperature distribution in GaN HEMT under gate injection at different times. (**a**) 10 ns; (**b**) 20 ns.

To investigate the specific damage locations of GaN HEMT under gate-injected HEMP, TCAD simulation is performed to analyze the current density and temperature distribution inside the device at different time instants, as shown in Figure 7 and Figure 8. The two moments correspond to Stage A–B and Stage B–C, as shown in Figure 6.

Under no HEMP injection, carriers, electric field, and current in the GaN HEMT are mainly confined within the two-dimensional electron gas channel, and the device remains in a stable state. When HEMP injects into the gate, the gate voltage stays low before 10 ns. Voltage drops mainly across gate-drain, leading to weak channel electric field and current densit; the device only heats mildly with no obvious variation. At 10 ns, rising pulse voltage sharply boosts the local gate-source electric field and triggers avalanche breakdown, which rapidly elevates current and temperature. At 20 ns, the pulse voltage approaches its peak value. The variation of the electric field becomes slower. Meanwhile, carrier velocity saturation limits further acceleration of hot carriers. Therefore, the temperature rise rate decreases, although the device temperature continues to increase.

**Figure 9:**
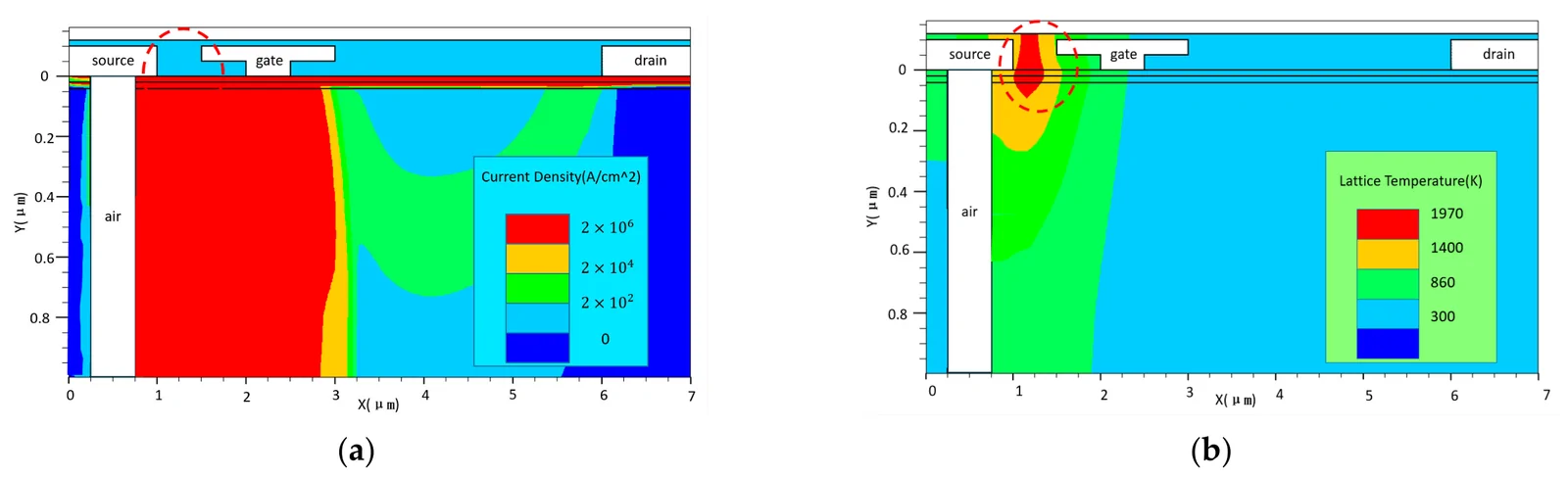
Current density and temperature distributions in GaN HEMT at the burnout instant. (**a**) Current density; (**b**) temperature.

As time progresses, the device’s internal temperature continuously climbs to the burnout threshold. The corresponding internal current density and temperature distributions at this critical moment are illustrated in Figure 9.

Figure 9a reveals that the peak current density mainly accumulates near the gate edge on the source side. This demonstrates that thermal accumulation renders the pillar-shaped surface region adjacent to the source-side gate edge the most vulnerable to damage, which aligns with the hotspot position displayed in Figure 9b.

#### 2.4.2. Influence of HEMP Waveform Parameters on GaN HEMT Damage Under Gate Injection

To investigate the influence of injection source variations on GaN HEMT damage, repeated simulations are performed with adjusted peak injection voltages and waveform parameters. Table 3 lists the damage power threshold and damage time under 20/500 ns waveform parameters for different injection peaks.

Curve fitting on the simulation results produces a semi-empirical formula of transistor thermal damage power for the established GaN HEMT model, as expressed in Equation (7).

(7) $$P=74.4120\cdot t^{-0.0508}$$

In the formula, P denotes the thermal damage power threshold of GaN HEMT in dBm, and t represents its thermal damage time in ns.

It can be observed that the thermal damage time of the device decreases with an increase in thermal damage power threshold. This is primarily attributed to energy accumulation. Higher thermal damage power speeds up energy accumulation in a short period, so the device rapidly reaches the critical damage temperature and incurs thermal failure quickly.

**Figure 10:**
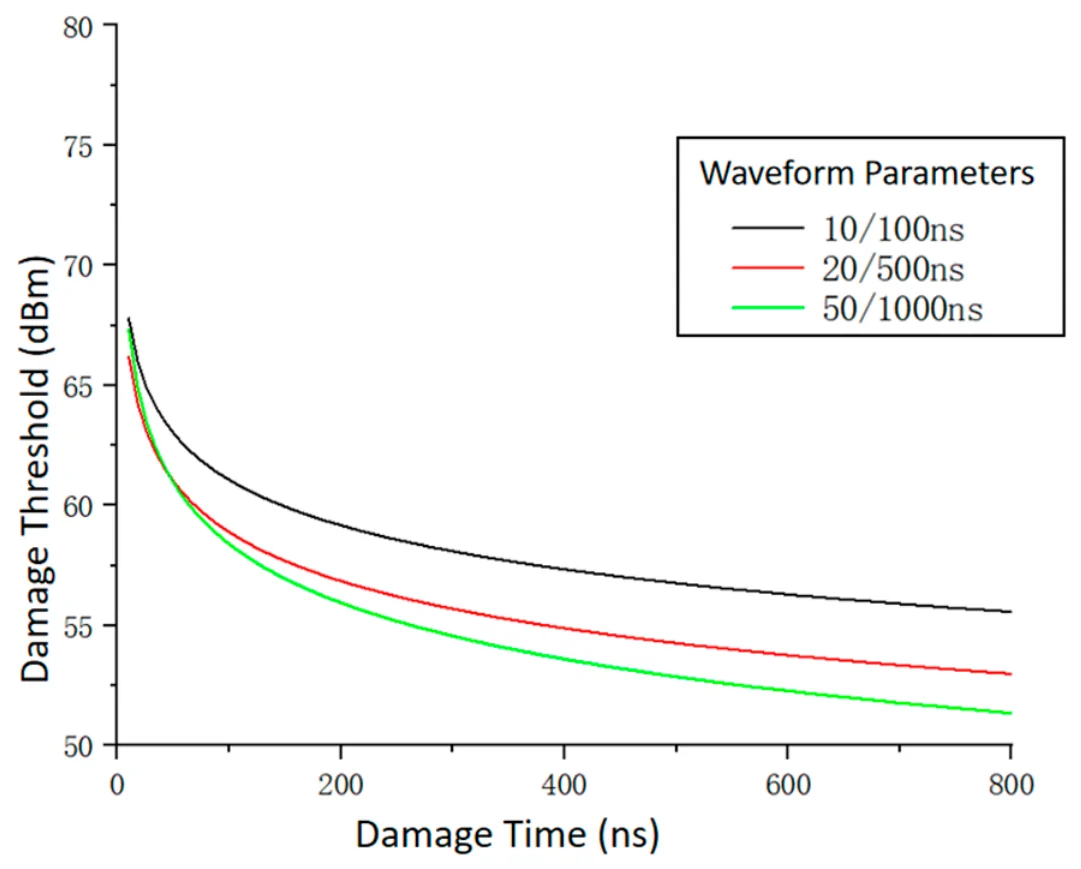
Damage threshold versus damage time curves under different HEMP parameters.

Waveform parameters are set to 10/100 ns and 50/1000 ns. The corresponding correlations among damage power threshold, damage time and injection peak for each parameter group are obtained but not displayed in detail. Fitted curves of damage threshold versus damage time for the three waveform conditions are compared together in Figure 10.

Distinct discrepancies in thermal damage time are observed for GaN HEMT under various HEMP pulse waveforms. When the power threshold exceeds 65 dBm, the internal electric field of the device is driven to the critical breakdown level in a shorter period to accelerate localized hotspot formation, and the shortest thermal damage time is thus documented for the 10/100 ns waveform. For power thresholds between 58 dBm and 65 dBm, the 10/100 ns waveform exhibits a fast rising edge yet rapid post-peak decay. Sustained heat accumulation is inhibited, and the initiation of damage is postponed, so the minimum damage duration is obtained with the 20/500 ns waveform in this interval. When the power threshold drops below 58 dBm, the effect of pulse duration on damage evolution is enhanced as pulse loading time increases, and the shortest damage time is therefore measured for the 50/1000 ns waveform.

In conclusion, the thermal damage time of GaN HEMT subjected to HEMP excitation is jointly determined by the rising-edge characteristics and duration of incident pulses. Device damage is more easily triggered by pulses with steeper rising edges at high power thresholds, whereas thermal accumulation and subsequent device failure are more likely to be produced by wide-pulse waveforms at low power thresholds.

## 3. Damage Analysis of GaN HEMT Power Amplifier Under HEMP

To investigate the damage effects of the GaN HEMT power amplifier exposed to HEMP, a GaN HEMT power amplifier circuit is constructed in this section, and HEMP signals are injected into the amplifier under normal operating conditions. The intrinsic relationships between device failure and pulse parameters are uncovered through an analysis of how HEMP waveform parameters affect the gain and damage threshold of the amplifier.

### 3.1. Design of the GaN HEMT Power Amplifier

Serving as a vital frequency band for communication systems, the L-band is extensively utilized in satellite navigation, radar detection and related applications. Accordingly, the power amplifier developed in this paper is designed to operate primarily within the L-band. The detailed specifications are listed in Table 4.

Using ADS to determine the static operating point through DC bias scanning, build a feedback network to achieve full-band stability, then obtain the optimal matching impedance through source/load pull testing, design the input and output microstrip matching circuits and the 1/4-wavelength bias network, and integrate them to form a complete power amplifier circuit. Upon completion of the design, transient-characteristic simulation is performed on the power amplifier circuit. A signal gain exceeding 15 dB across the designed frequency band is obtained, with a gain of 15.26 dB at the center frequency of 1.8 GHz. For an input power of 30 dBm, an output power of approximately 41.8 dBm and a power-added efficiency of around 67% are achieved for the power amplifier over the target band.

**Figure 11:**
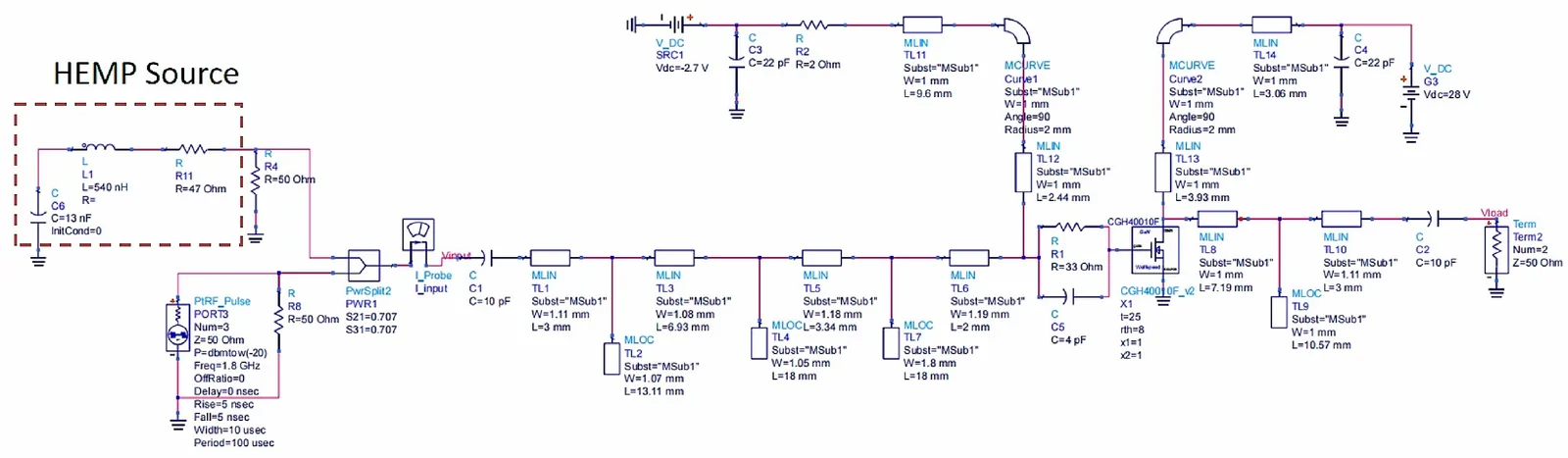
ADS simulation circuit of power amplifier response to HEMP.

To investigate the impacts of HEMP on the normal operation of power amplifiers, the HEMP source is constructed based on the RLC equivalent circuit presented in Section 2.3.1, and the operating small signal and HEMP are simultaneously injected into the input terminal of the power amplifier via a power combiner [19,20,21,22]. In the simulation, the parameter values of the RLC circuit are adjusted to obtain HEMP with different waveform parameters and analyze their effects on the power amplifier under normal operating conditions. The integrated simulation circuit is illustrated in Figure 11.

Power amplifier gain is defined as the ratio of output power to input power. A positive gain under normal operation means the input signal is amplified effectively and the circuit operates as a normal power amplifier. When HEMP is injected, negative gain indicates severe performance degradation and total loss of amplification function. This section therefore uses negative power amplifier gain as the criterion for performance degradation or functional failure to assess how HEMP injection affects the operating performance and functional sustainability of the power amplifier.

### 3.2. ADS Simulation Results and Discussion

To analyze the influence of HEMP pulse waveform parameters on power amplifier damage, the HEMP generation circuit is adjusted to produce 10/100 ns, 20/500 ns, and 50/1000 ns waveforms, respectively. By comparing the results under different waveform parameters, the effects of pulse rise time and pulse width on the response characteristics of the power amplifier are investigated.

**Figure 12:**
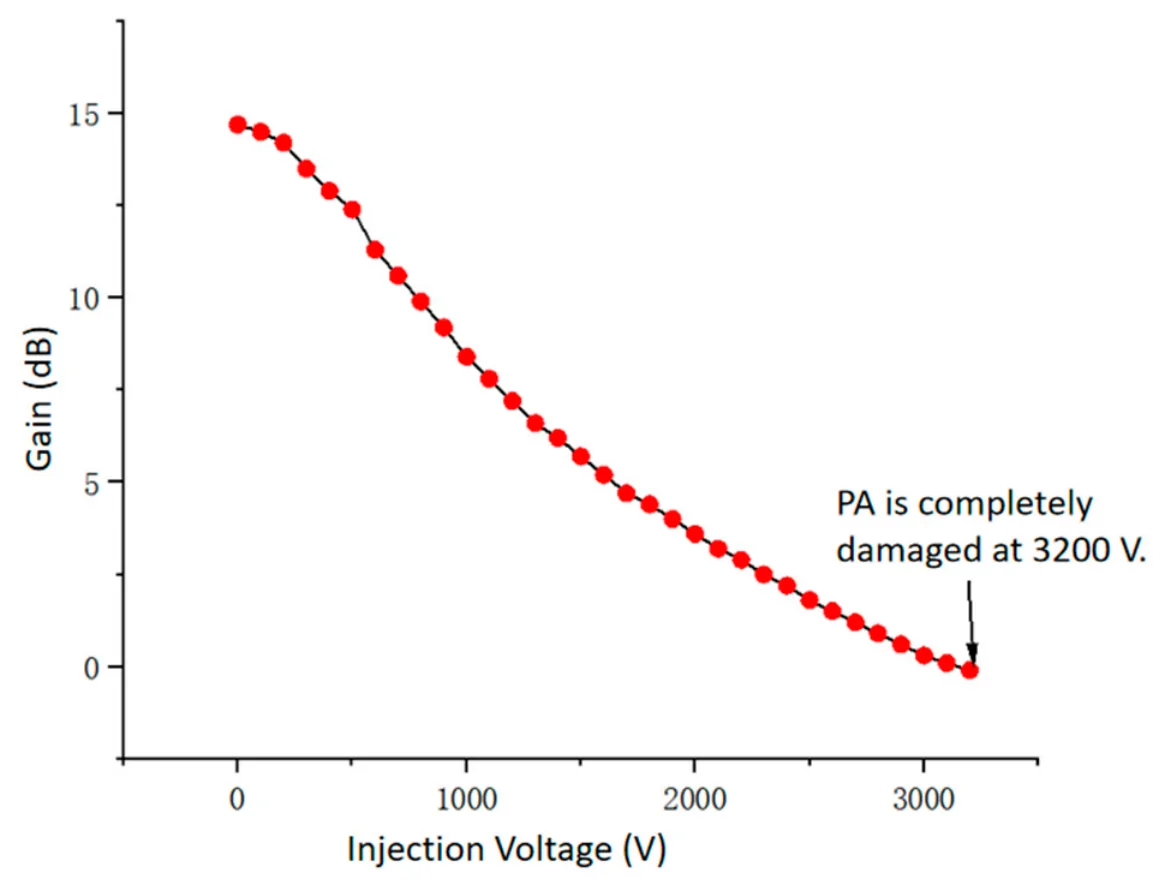
Gain variation of power amplifier versus 20/500 ns HEMP injection voltage.

Figure 12 shows the gain variation of the power amplifier as a function of the 20/500 ns HEMP injection voltage. Table 5 presents a comparison of the damage thresholds of the power amplifier under the three HEMP waveform conditions.

Under different HEMP waveform conditions, the damage threshold of the power amplifier shows a clear pulse-width dependence. As the HEMP duration decreases, the damage voltage threshold increases significantly, whereas it decreases as the pulse width becomes larger.

The simulation results reveal that the damage process of the power amplifier is not governed solely by peak power. Instead, it is more closely related to the cumulative energy deposition process. For wider pulses, although the peak voltage is lower, the longer interaction time allows the device to absorb electromagnetic energy over an extended period, making it easier to reach the critical condition for damage. In contrast, for narrower pulses, the reduced energy deposition time requires a higher peak voltage to compensate for the insufficient duration, ensuring that the injected energy reaches the failure level of the device. Therefore, the observed decrease in damage voltage threshold with increasing HEMP pulse width in Table 5 can be reasonably explained from the perspective of energy accumulation effects.

## 4. Experimental Study on HEMP Injection Damage of GaN HEMT Power Amplifier

Limited by the constraints of circuit simulation, only the overall performance degradation process of the power amplifier can be observed after HEMP injection, while the internal damage of the GaN HEMT cannot be captured. Accordingly, damage effect experiments are further carried out in this section based on the aforementioned power amplifier simulation.

The experiment used a total of four GaN HEMT chips and one power amplifier module. Due to differences in transistor batches, there were slight variations in the standard current and small differences in small-signal gain among the different transistors, but these differences did not affect the conduction of the damage injection experiment.

The four GaN HEMT chips are denoted as Chip 0 to Chip 3. Chip 0 is selected as the reference standard. Chips 1, 2, and 3 are adopted for the injection experiment.

### 4.1. Design of Injection Experiment

**Figure 13:**
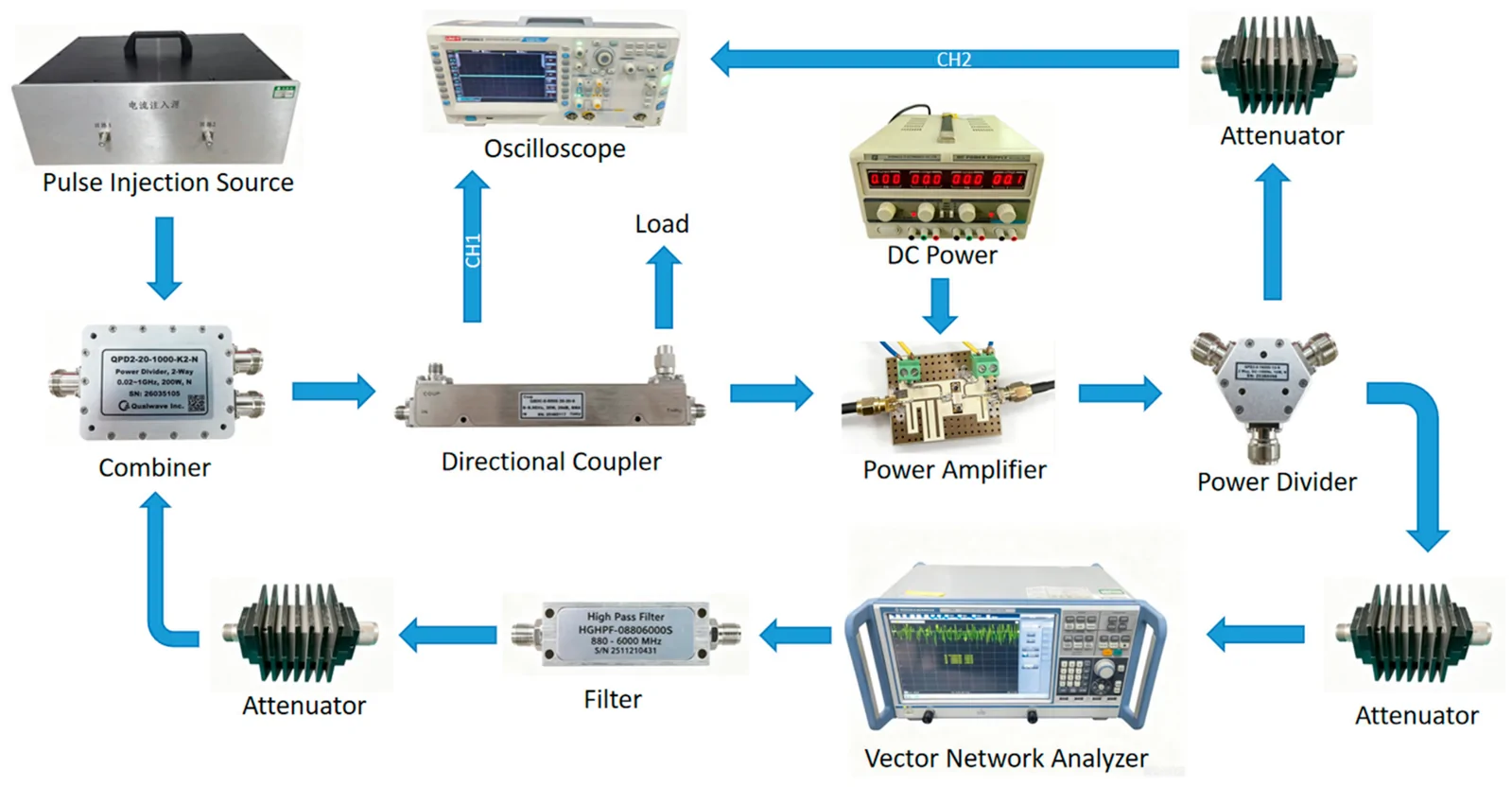
Schematic of HEMP injection experiment platform.

Figure 13 shows the schematic diagram of the experimental platform for the HEMP injection experiment. The oscilloscope used in the experiment is Model MDO3102, manufactured by Tektronix. The vector network analyzer is Model ZND from Rohde & Schwarz. The DC power supply is Model DF1731SLL3A, produced by Zhongce Electronics. The pulse injection source is a multi-parameter pulse-current injection source developed by Puosi Electric Technology Company. The combiner adopts model QPD2-20-1000-K2-N with an attenuation of 3.5 dB. The directional coupler, model QSDC-0-8500-30, operates over DC-8.5 GHz, provides a 20 dB coupled output, and features an insertion loss of 0.8 dB. The power divider, model QPD2-0-18000-10, operates over DC-18 GHz and has an attenuation of 7 dB. The attenuator adopts the model TS50 and has an attenuation of 20 dB. The filter, Model HGHPF-08806000S, has a passband of 0.88–6 GHz. The total attenuation introduced by the RF cables throughout the test setup is taken as 1.5 dB.

Similar to the power amplifier simulation described above, the experiment involves two signal paths: one is a high-power pulse injection path, in which the HEMP signal generated by a pulse-current source is injected into the power amplifier under test via a power combiner and a directional coupler. The signal coupled out through the coupler is monitored by an oscilloscope to measure the injected power, while the power amplifier’s output signal is measured by an oscilloscope on another channel after passing through a power divider and an attenuator. The other path is the small-signal monitoring path. The small-signal output by the vector network analyzer passes through a filter and an attenuator before being injected into the power amplifier via a power combiner and a directional coupler. The output signal from the power amplifier is split by a power divider, attenuated, and returned to the vector network analyzer for reception. This path is used for real-time, online measurement of gain variations in the power amplifier, and gain monitoring is used to determine whether the power amplifier has sustained damage [23,24].

The experiment is performed at room temperature, approximately 25 °C, with a relative humidity of about 50%. During the experiment, the pulse injection source generates HEMP of different amplitudes with a 20/500 ns waveform. For each HEMP injection, the oscilloscope voltage and the |S_21_| measured by the vector network analyzer are monitored in real time to confirm successful injection. If the measured |S_21_| remains unchanged after HEMP injection, indicating no significant performance degradation of the power amplifier, the voltage amplitude of the injected pulse is increased step by step. Device functional degradation is confirmed once an irreversible change in |S_21_| is detected. This threshold provides an objective termination condition for the experiment and is adopted to systematically evaluate the damage tolerance of the power amplifier under HEMP.

### 4.2. Results and Discussion

**Figure 14:**
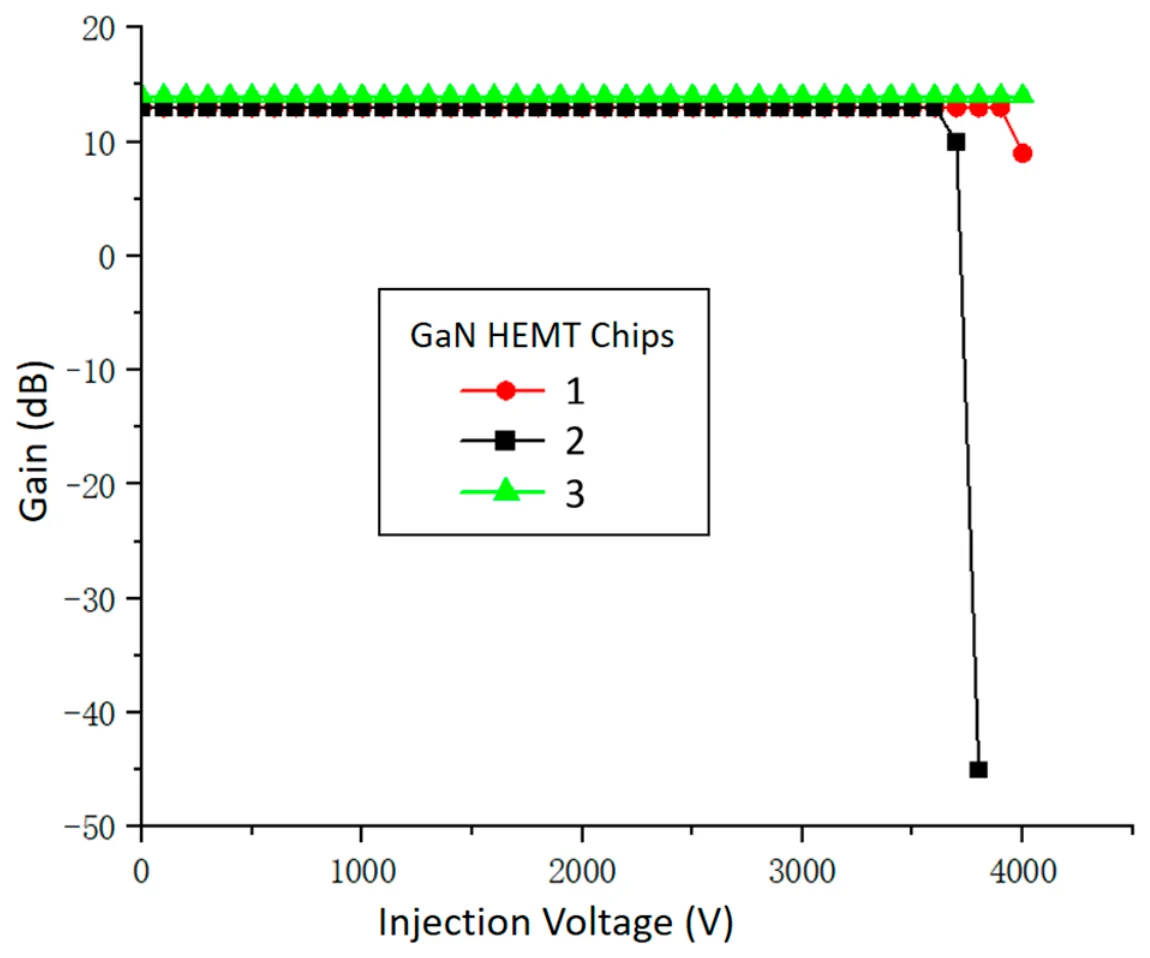
Gain of power amplifier versus HEMP injection voltage.

The HEMP injection platform in Figure 13 is built and tested in triplicate. Considering the operating frequency ranges of the power combiner and the power amplifier, the magnitude of the forward transmission coefficient |S_21_| at 1.6 GHz is selected as the criterion for evaluating power amplifier failure. The pulse injection voltage is increased in increments of 100 V. After each injection, the output waveform of the directional coupler is observed to verify successful pulse injection, while the vector network analyzer is used to determine whether any device damage occurs. The measured variation in the power amplifier gain as a function of the HEMP injection voltage is presented in Figure 14.

Taking Chip 2 as an example, the gain remained stable before the injection voltage reached the damage threshold. When the injection voltage increased to 3700 V, the small-signal gain decreased by 3 dB, indicating that the power amplifier had been damaged, although it still exhibited a gain of 10 dB and remained partially functional. As the injection voltage is further increased to 3800 V, the gain dropped to −44 dB, indicating complete failure of the power amplifier.

The experimental results indicate that, under HEMP injection, the damage threshold of this power amplifier model is 3800 V. The primary reason why the gains of the other samples did not decrease at 3800 V is attributed to sample-to-sample variations. Although all samples may begin to exhibit gain degradation at this injection voltage, differences in fabrication batches, manufacturing processes, and device characteristics lead to variations in their sensitivity to HEMP. Consequently, the samples exhibit different damage thresholds.

A difference is observed between the experimental gain trend shown in Figure 14 and the simulated trend shown in Figure 12. This difference is attributed to the self-recovery capability of the device, which originates from the trapping effect. Within the GaN HEMT, numerous localized defect energy levels are introduced by crystal defects. When current is injected, carriers in the channel are captured by these traps, and an additional electric field is generated inside the device, which repels the two-dimensional electron gas. As a result, the channel carrier concentration is reduced, and the channel resistance is increased, which macroscopically manifests as performance degradation, including reduced drain current and gain compression [25,26]. This degradation becomes more pronounced as the injection voltage is increased. After the pulse is terminated, the trapped carriers are released from the traps through thermal excitation or tunneling effects, and the electrical performance of the device is partially or fully restored. The recovery time depends on the trap energy level depth; shallower traps recover faster, with typical time constants in the microsecond to millisecond range. In simulations, the time step can be reduced to nanosecond or even picosecond levels, so short-term gain degradation can be captured. In experiments, due to the limitations of the measurement equipment, only short-term gain fluctuations can be observed. Since the present study is focused on irreversible hard damage, the gain values measured during the experiments are those obtained after the device has stabilized.

**Figure 15:**
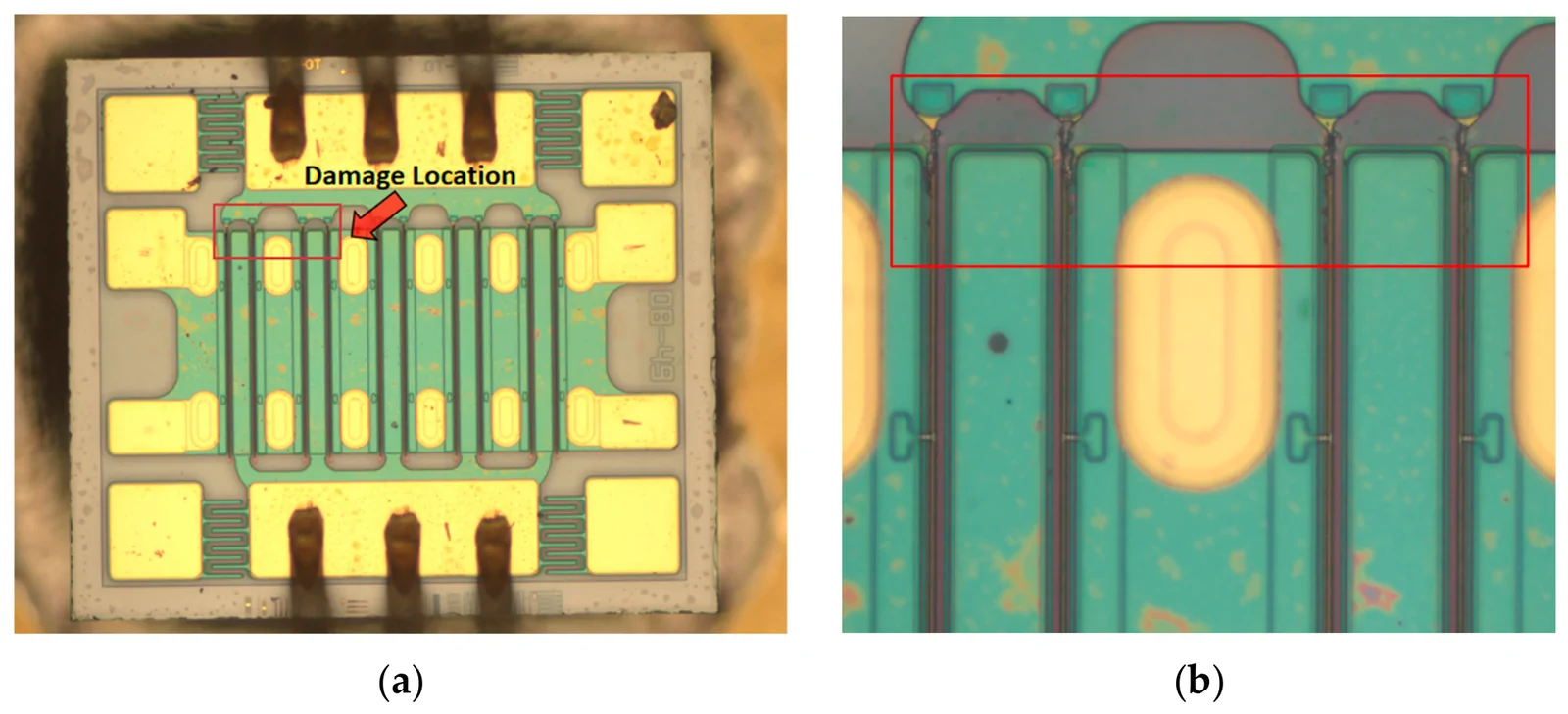
Morphology of failed Chip 2. (**a**) Internal view of failed Chip 2; (**b**) partial enlarged view of failed Chip 2.

To further investigate the failure mechanism of the GaN HEMT under HEMP injection and identify internal abnormalities within the device, the failed Chip 2 was decapsulated using a mechanical decapsulation method. The surface morphology of the decapsulated failed sample and the marked damage location are illustrated in Figure 15a, and a local magnified view of the damaged region is provided in Figure 15b.

The results show that the damage occurred in the channel between the gate and source. Pulse injection caused severe burnout of the gate–source channel within the chip. Because the entire gate–source channel of the GaN HEMT was destroyed, the GaN HEMT power amplifier completely lost its gain.

### 4.3. Comparison Between Simulation and Experiment

A comparative analysis was conducted to verify the consistency of the established GaN HEMT device model and power amplifier circuit simulation model with experimental results under HEMP injection. Comparisons are made between the simulated GaN HEMT damage positions under HEMP injection in Section 2.4, the simulated power amplifier damage thresholds under HEMP injection in Section 3.2, and the experimentally measured damage thresholds and failure positions presented in Section 4.2. The comparison results are summarized in Table 6.

As shown in Table 6, the combiner introduces 3 dB attenuation for HEMP in the simulation. The actual injection voltage is 2265 V when the pulse injection source voltage reaches 3200 V. In the experiment, the combiner also introduces 3 dB attenuation for HEMP. The directional coupler and RF cable introduce approximately 0.8 dB and 0.5 dB attenuation for HEMP, respectively. This yields a total attenuation of about 4.3 dB for HEMP before it reaches the power amplifier, and the actual injection voltage is approximately 2316 V at a pulse injection source voltage of 3800 V. Considering the attenuation, the actual injection voltage is close to 2300 V, with an acceptable error for both operating conditions when the power amplifier reaches the damage threshold induced by HEMP.

Regarding damage position, TCAD simulation reveals that damage emerges in the gate-source channel of the GaN HEMT. Cross-section analysis of the tested chip confirms that the failure region on the GaN HEMT chip within the power amplifier is also located in the gate-source channel.

In conclusion, the effective injection voltage corresponding to the damage threshold of the GaN HEMT power amplifier predicted by simulations is highly consistent with experimental data, and the simulated damage position of the GaN HEMT matches the cross-sectional observation of experimental samples. Excellent consistency between simulation and experiment is thus verified.

## 5. Conclusions

In response to the severe damage effect of power amplifiers under strong electromagnetic pulses, the performance of the radio frequency transmission system can be significantly affected. In this paper, a physical model of GaN HEMT is first established, and a gate injection simulation is implemented to capture the damage evolution process of GaN HEMT. It reveals the influence rule of pulse parameters on the electrical and thermal effects of the device. Subsequently, a GaN HEMT power amplifier model based on CGH40010F below 6 GHz is built to conduct HEMP injection simulations; the results demonstrate that the damage evolution of the power amplifier is closely associated with the accumulation of injected energy of different HEMP pulse parameters. Furthermore, gate composite pulse injection experiments are performed with a HEMP injection system. Experimental results and chip cross-section observations indicated that power amplifier failure arises from the damage of the GaN HEMT, and the measured damage thresholds as well as device damage locations are basically consistent with simulation results. The conclusions above offer a theoretical foundation and data reference for damage evaluation of GaN devices exposed to extreme electromagnetic environments and provide important guidance for the protection design of radio frequency systems working in complex electromagnetic environments. This research can be applied to the damage assessment in other types of GaN power amplifiers and can guide the design of protective circuits. Based on the specific application scenarios, different coupling directions of a strong electromagnetic pulse can be used to conduct further analysis of the coupling effects and damage assessment of the circuit.

## Figures and Tables

**Table 1:** Key structural parameters of the GaN HEMT.

Parameter	Unit	Value
Source-to-Gate Spacing	µm	1
Gate Length	µm	0.5
Gate-to-Drain Spacing	µm	3.5
Passivation Layer Thickness	nm	120
AlGaN Barrier Layer Thickness	nm	20
GaN Channel Layer Thickness	nm	20
GaN Buffer Layer Thickness	µm	0.98
AlGaN Barrier Layer Doping Concentration	cm^−3^	10^17^
GaN Channel Layer Doping Concentration	cm^−3^	10^16^
GaN Buffer Layer Doping Concentration	cm^−3^	10^15^

**Table 2:** RLC parameters for different HEMP waveforms.

Waveform Parameters	Capacitance (nF)	Inductance (nH)	Resistance (Ω)
10/100 ns	1.3	333	74
20/500 ns	13	540	47
50/1000 ns	23.3	1375	50

**Table 3:** Damage power threshold versus damage time for GaN HEMT at 20/500 ns injection.

Injection Voltage/V	Damage Power Threshold/dBm	Damage Time/ns
200	59.03	100
250	60.96	49
300	62.55	28
350	63.89	20
400	65.05	15

**Table 4:** Design specifications of power amplifier.

Performance Specifications	Specification Range
Frequency Range (GHz)	1.6~2.0
Power Gain (dB)	≥15
Saturated Output Power (dBm)	≥40
Efficiency (%)	≥60
Input Return Loss (dB)	≥10
Power Supply Requirements	*V_ds_* = 28 V, *V_gs_* = −2.7 V
Operating Temperature (℃)	−55~125

**Table 5:** Damage threshold variation of power amplifier with HEMP waveform characteristics.

Rise Time/ns	Full Width at Half Maximum/ns	Damage Voltage Threshold/V
10	100	7200
20	500	3200
50	1000	2100

**Table 6:** Damage comparison between simulation and experiment.

Injection Mode	Injection Object	Damage Threshold/V	Actual Injection Voltage/V	Damage Location	Simulation/Experiment
Gate Single Pulse	GaN HEMT	200	200	Gate-source channel	TCAD Simulation
Gate Composite Pulse	Power Amplifier	3200	2265	Unidentified	ADS Simulation
Gate Composite Pulse	Power Amplifier	3800	2316	Gate-source channel	Experiment

## Data Availability

The raw data supporting the conclusions of this article will be made available by the authors on request.
